# Repertory of the Back

**Published:** 1898-11

**Authors:** E. H. Wilsey

**Affiliations:** Parkersburg, W. Va.


### REPERTORY OF THE BACK.

E. H. Wilsey, M. D., Parkersburg, W. Va.

(Continued from October No., p. 452.)

### SCAPULÆ.

**Chest**, stitches from chest to scapulæ, Ox-ac.
— shooting from inner angles of left scapula to front of chest, Naja.
— cutting from point of left scapula through chest to middle of sternum, Thuja.

**Chill,** pain under scapulæ with chill, Sang.
— pain along inner border of right scapula, with heat and chills alternating and extending to kidneys, where there is heat and pain, Sang.

**Chills** or creeping along back between scapulæ; hard pressing pain in fourth cervical vertebræ, *Lac-def.*

**Chilliness,** cutting and stitches in scapulæ, Alumina.

**Clavicle,** pain in left scapula, also under clavicle and down arm, with feeble pulse, Aspar.

**Clucking** in muscles of left scapula, Asaf.

**Cold** pressure on lower end of left scapula, Plat.
— tearing in left scapula as from a cold, Sepia.

SCAPULÆ.

**Cold** pain in scapulæ, with sensation as if a small piece of cold iron pressed against it, Phyto.

**Continuous** dull, heavy pain in left scapula, *Cund.*

**Contractive** pain between scapulæ when standing, China.

— pain from superior angle of scapulæ, drawing shoulders back tight as if bones would be crushed, Medor.

**Cough**, pressing pain in scapulæ with cough, Corallium.

— pain below left scapula with cough, *Sticta.*

— with pain in sternum, darting back through to between scapulæ, Kali-b.

**Coughing**, scapulæ pain when coughing, Sul.

— pain between scapulæ when coughing, Stram.

**Cracking**, frequent returning pressing cracking pain on left posterior side of neck near scapulæ; not changed by any motion, Spong.

— in scapulæ on lifting arm, Anac.

**Cramplike** drawing pain in muscles of neck, down into the scapulæ in evening after lying down and in the morning; worse by stooping or turning head to left, Ant-c.

— pinching in intercostal border of right scapula, with a kind of a pressing stitch from left side of occiput to forehead, Chelidon.

— sudden pain in left scapula, Baryta-c.

**Crampy** pain cutting through left side of chest and scapula, *Nat-m.*

**Cutting** in left scapula, Diosc.

— in left scapula; better by motion, Merc-i-fl.

— under left scapula, Thuja.

— under left scapula after dinner, with pressure in stomach, Hyperi.

— cramplike pain through left chest to scapulæ, *Nat-mur.*

**Darting**, from left scapula to shoulder and mammæ, Grat.

**Deep**, sharp pains in lower portion of left scapula at 1 P. M., followed by sharp pain through centre of right lung, Diosc.

— seated pain in right scapula, preventing motion, Ailanth.

### SCAPULÆ.

**Deep** seated pain below point of scapula, worse toward left side, worse when sitting, especially when riding, Fluor-ac.

**Dinner,** shooting in right scapula after dinner, Nat-c.

— heavy pain under left scapula after dinner, Physos.

**Dislocation**, pain in scapulæ, as from dislocation, China.

**Dorsal,** violent pain in upper three dorsal vertebræ, extending through scapulæ, *Kalmia.*

— pain in dorsal region of left scapula, Cajup.

**Drawing,** in scapulæ, Sarsa.

— between scapulæ, Thuja,

— along base of left scapula, Senega.

— and tearing in left scapula, partly toward back and partly toward shoulder, Stann.

— between scapulæ at night during menses, Sil.

— in calves and scapulæ, Nitr-sp-d.

— in right scapula, worse by motion, Sul.

— compressive in region of scapulæ, Ran-scl.

— in nape, extending to right scapula, Xanthox.

— and pressing in scapulæ, Kali-c.

— pressure in right scapula, then in left, Tell.

— in and near right scapula in evening, Lyco.

— and tearing in the right scapula, compelling him to breathe deeply, Nat-m.

— pressure near spine beside left scapula, at times into nape, Sepia.

— jerk-like in right scapula, extending to hand, Plat.

— and tearing in scapulæ, *Caustic.*

— pain in right shoulder, Euphor., Nat-c.

— contractive pain from superior angle of scapulæ, drawing shoulders back tight, as if bones would be crushed, Medor.

— pain in nape of neck and scapulæ, Sul.

— pain in right scapula evenings, on going to sleep, Sul.

— paroxysmal pain in scapulæ, then in nape and head, when she became dizzy, Sil.

— painless drawing in left scapula, *Squilla.*

### SCAPULÆ.

**Drawing** pain on inner border of right scapula, Carduus.
— pain on outer border of left scapula, Carduus.
— pain from right scapula down back when driving, Carduus.
— pain in scapulæ, Cham.
— pressive pain between scapulæ, Calend.
— pain between scapulæ, necessitating lying down, *Ars.*
— aching pain in shoulder joints and in left scapula, Ptelia.
— piercing pain close under left scapulæ for a few moments while sitting, *Millef.*
— pain between scapulæ, *Calc-c.*, Diosc.
— pain between scapulæ, drawing to small of back, *Drosera.*
— a long breath; worse pain under right scapula, Jug-cin.
— internally in region of right scapula, as if nerve and vessels were made tense, *Colocy.*
— pain in right scapula, Con.
— rheumatism passing from nape of neck to left shoulder, then into scapula, evening while walking out, Borax.
— tearing on outer border of right scapula, Dulc.
— tensive in right scapula, Sil.
— sharp stitches with fine drawing and sensation of heat in scapulæ, Mur-ac.
**Dull** pain under right scapula, near spine, *Chenop-a.*
— heavy continuous pain in left scapula, Cund.
— pain between scapulæ, *Phos.*
— pain along inner edge of left scapula, worse from breathing, *Sang-c.*
— pain under right scapula, Indium.
— sticking pains in shoulder joints, Staph.
— pain at inner angle of left scapula in evening, and at inferior angles of both in morning, Chromic-ac.
**Eructation**, sharp lancinating pain close to upper part of right scapula for many days, most painful during eructation, Zinc.
**Eruption**, on scapula without itching, only pains when touched, Phos-ac.

SCAPULÆ.

**Evening,** drawing pain in right scapula in evening on going to sleep, Sul.
— pain in right scapula afternoon and evening, Ver-v.
— dull pain at inferior angle of left scapula in evening, and superior angle of both in morning, Chromic-ac.
**Fleas,** a vesicle forms on left scapula as from fleas, Amm-c.
**Formication** in left scapula, Sil.
**Fracture,** tearing pain as from a fracture in the scapula, with stiffness of back and neck, Nat-m.
**Gnawing** on scapula, Phos-ac.
— between scapulæ, Nat-c.
— and stitches in scapulæ, Alum.
**Gurgling** and rolling sensation in right scapula, feeling chilly, Tarax.
**Hawking,** pressive, now shooting pain beside right scapula in deglutition and in hawking, Caust.
**Itching** on and between scapulæ, Alumina.
— on left scapula, with small pimples after scratching, Bary-c.
— over right scapula, and over left ribs, Diosc.
— on right scapula, Anagallis.
— stitches on right scapula, Calc-c.
— stitch near right scapula, better by scratching, Cann-s.
— and burning on spot below scapula (right), Pallad.
— violent on left scapula, *Formica-r.*
**Jerking** pain in left scapula, *Squilla.*
**Knot,** small inflamed, very sensitive knot on right scapula, not passing to suppuration, Amm-c.
**Lancinations** constant on border of left scapula toward the axilla so violently that she was startled; attended with rise of heat to head, Zinc.
**Lancinating** tension in right scapula, Carbo-an.
— pain in the back, extending to right scapula, Lyco.
**Mammæ,** pain in neck and left scapula, seemingly from left mammæ, Lil-tig.
**Margin,** pain at outer margin of left scapula, *Chelid.*

SCAPULÆ.

**Micturition,** during micturition a sensation as if something were running internally in right scapula, Hepar.

**Morning,** tearing in right scapula in morning, *Kali-c.*

— pressure on right scapula in morning in bed, Nat-sul.

— pain in right scapula morning when waking, Ran-b.

**Motion**, tensive pain between shoulders during rest and motion as from an adhesive plaster near inner margin of right scapula, Zinc.

— deep-seated pain in right scapula, preventing motion, Ailan.

— severe pain under lower angles of left scapula; worse from motion or breathing; can't take a full breath, Cup-ars.

— pain between scapula and over region of left pelvis during rest, with painful jerking on motion, Sul.

— pain as from a sprain in right shoulder only on motion, Staph.

— violent pressive pain in left shoulder joint; not better by any motion, Staph.

**Moving**, sprained pain in right scapula on moving arm, Sul,

— pain in right scapula, extending across shoulders to clavicle; worse moving arm or head, better by pressure, Mag-m.

— pain between scapulæ; worse by moving arms, Naja.

— pain in back under scapulæ; worse by moving, *Apis.*

— pain below right scapula; worse evenings after exertion by deep inspiration and by moving right arm, *Ruta.*

— arm or letting it hang down; worse pain in scapula, Ign.

**Movements,** pressing pain between scapulæ when stepping hard or on other movements which concuss the chest, Senega.

**Muscular** pains about lower margins of scapulæ in women who follow sedentary employment, Ran-b.

**Muscles,** rheumatic pain in scapular muscles; they feel tense and swollen, motion is difficult, *Mez.*

**Neck,** pain in right scapula, running up to neck, Still.

— pain in scapulæ and in back, extending into neck with tension, *Coloc.*

— neuralgic pain in neck and between scapulæ, Tabac.

SCAPULÆ.

**Neck,** pain in neck and left scapula, seemingly from left mammæ, *Lil-t.*

**Neuralgic** pain over right scapula, Anac.

**Oppression,** and pressing in right scapula, extending through thorax to sternum, *Chelid.*

**Outer** margin of left scapula, pains, *Chelid.*

**Over** left scapula, a pain, Merc-i-fl.

— left scapula, an indistinct murmur and purring sound, Sumbul.

— right scapula, a neuralgic pain, Anac.

— scapula, a pain, as if beaten, Ars-alb.

— itching over right scapula and left ribs, Diosc.

**Overcoat,** a sore pain in left scapula, worse on taking off overcoat, Cyclam.

**Paralytic,** tearing across scapulæ, with sticking and paralytic pain in arm, Cycl.

— pain in scapulæ, as if bruised when standing or sitting, better by lying, Asar.

— pain between scapulæ, and in neck in morning, Nat-c.

**Paroxysmal** drawing pains in scapulæ, then in neck and head when she became dizzy, Sil.

**Peg**, pain, as from a peg driven into left scapula, Phos.

**Piercing,** drawing pain close under left scapula for a few minutes, while sitting, Millef.

**Pimples,** on top of shoulder, with violent itching and burning after scratching, Kali-c.

**Pinching** and shooting in both scapulæ, Kali-c.

— pain in left scapula, Euphorb.

— after midnight, on motion in bed, Merc.

— of flesh of right scapula, Amm-m.

**Plaster,** sensation, as from a pitch plaster, near inner border of right scapula, Zinc.

**Point,** a pain beneath the point of right scapula, Lauroc.

**Pointed,** stitches painful from left scapula to axilla, Oleum-an.

**Pressing** pain in scapulæ, with cough, Corrallium.

— pinching, cramp-like pain in internal border of right scapula,

SCAPULÆ.

with a kind of pressive stitch from left side of occiput to forehead, Chelid.

**Pressing** in upper scapular region, with stiffness on sitting still, pain worse on beginning to move, Indium.

— pain under right scapula, and between scapulæ, Lyss.

— between scapulæ, when stepping hard, or on other movements which concuss the chest, Senega.

— and oppressive pain in right scapula, extending through thorax to sternum, *Chelid.*

**Pressive** pain beneath the scapulæ in the evening, Sul.

— violent pain in left shoulder joint, not better by any motion, Staph.

— wandering pains in dorsal muscles, below scapulæ, Brom.

— pains between scapulæ, which seem to come from posterior walls of stomach, Arn.

— drawing pain between scapulæ, Calend.

**Pressure,** pain in right scapula, extending across shoulders to clavicle, worse on moving arm or head, better by pressure, Mag-m.

— pain between scapulæ, as from a sprain and extending forward into chest, Petrol.

— pain below right scapula, extends over a spot as large as the palm, *Ruta.*

— tensive upon a small spot upon the back by border of right scapula, Zinc.

— in left scapula, Kali-c.

— on right scapula, Aur-m-nat.

— in scapulæ and nape of neck, *Coccul.*

— on right scapula in morning in bed, Nat-s.

— sharp on upper border of right scapula and clavicle, Bism.

**Pricking** and burning in left scapula, Fluor-ac.

**Prickling** in left scapula, extending to the left shoulder, Ginseng.

— tingling in region of left scapula at 2 P. M. with lameness of muscles, Apoc-can.

SCAPULÆ.

**Pulling** in the scapulæ, Copaiva.

**Pulsating** undulating in a spot below left scapula in P. M., causing a jerk through the whole body and forcing him to hold his breath with sensitiveness to touch, he was forced to stop speaking because the pain took away his breath, Sul.

— and beating in upper edge of left scapula, Kali-c.

**Quivering** in skin of scapula, Sil.

**Raising,** pain in left scapula, worse by raising left arm; between scapulæ and in lower part of chest, Clematis.

**Rawness** in right scapula during repose, Colocy.

**Rheumatic** sensation in left scapula when writing, Carbo-v.

— pain in right shoulder, Iod.

— pain in right scapula and left wrist, Asclep-t.

— pain between scapulæ, Aspar., Bry., Calad., *Camp.*, Lob-i., Lyco-vir., Mag-s., Ran-b., Rhod., Rhus.

— pain drawing in nape of neck extending into left shoulder, then into scapulæ, evening when walking in open air, Borax.

— pain in left scapula, Graph., Lyco.

— pain in scapulæ, Valer.

— pain in right scapula, Ambra, *Mez.*

— pain between scapulæ, better by warmth and worse by cold, Rhus.

— pain in upper left scapula after the usual bath, Carbo-v.

— pain in scapulæ muscles, they feel tense and swollen, motion difficult, *Mez.*

— pain between scapulæ, can hardly turn in bed, feels beating of pulse when lying down, Calad.

— pain in back between inferior angles of scapulæ, Bry.

— pain in scapular region, *Carbo-v.*

— pain in left scapular region so he could not raise arm to his head, *Lyco.*

**Rheumatism** of left scapula and nape, worse turning to opposite side, Mez.

### SCAPULÆ.

**Rubbing**, painful tension between scapulæ, better by rubbing, Carbo-an.

**Running**, sensation during micturition of something running internally in right scapula, Hepar.

**Seizing**, sensation as if something were seizing her firmly by the scapula, when lifting and carrying with both hands, Phos.

**Sensation** of pain on inner surface of scapula, *Cicuta*.

**Severe** pain under the lower angle of left scapula, worse from breathing or motion, cannot take a full breath, Cup-ars.

— pain in or near the head of right scapula, Bad.

— pain between scapula, better by inclining forward, worse from inspiration, it gradually passes around in front to ninth or tenth ribs, Nat-ars.

— pain under the left scapula, Indium., Iod.

— pain about superior margins of left scapula, Jalap.

**Sharp** pain under left scapula near axilla, Lappa.

— pain in angle of scapulæ, Iodof.

— pain below left scapula on inspiration, Codein.

— pains from under point of left scapula through to left breast, Aletris.

— alternating dull aching, and sharp pain beneath right scapula, at its lower or inferior angle, Bry.

— pain in left scapula suddenly, Merc-i-fl.

— pain through right shoulder to scapula, Comocladia.

— pain under left scapula, Daphne.

— deep pains in lower portion of left scapula at I P. M., followed by sharp pains through centre of right lung, Diosc.

**Shooting** pain in right scapula, *Phos.*

— bruised pain in right scapula during motion, perceptible even in the chest, Kali-c.

— and other pains between shoulders and along borders of scapulæ, has to straighten up to be relieved, Bov.

— violent pain as from a sprain in left scapula, extending into chest, Kali-c.

— pain under left scapula, *Calc-c.*

SCAPULÆ.

**Shooting** pain in left scapula when resting on one's left arm, Sul.

— violent shooting in lower part of right scapula below the axilla, Mur-a.

— in top of the shoulder at times with itching, Nat-c.

— fine pressing on the lower edge of right scapula, Mur-ac.

— in left scapula, Sepia.

— on right shoulder soon changing into straining, Mur-ac.

— and pressure, tearing in back near right scapula, Kali-c.

— in region of left scapula, from 11.30 till 12 M., Formica.

— in right scapula, electric from left to right, Bell.

— frequent shooting in right scapula, *Sil.*

— obtuse in left scapula, Kali-c.

— fine shooting in scapulæ extending into side and chest only when sitting, on walking fast it ceases, when walking slowly, chiefly in P. M. and evening, Sepia.

**Sore** in left scapula, worse by taking off overcoat, Cyclam.

— at point under left scapula, Papaya-vul.

— as if in left scapula when at rest, Coloc.

— along inner border of right scapula, Asar.

— and burning sensation in region of left scapula, worse by inspiration, pressure, and reclining, Carduus.

— in region of scapulæ, Lil-tig.

— intermittent sore sensation on outer border of right scapula, Plat.

**Soreness** at lower cervical vertebræ and under right scapula, Nat-ars.

— extends over right scapula at 9 P. M., Diosc.

— sensation of pressure on outer border of right scapula, Platinum.

**Spot** painful in left scapular region, Onos.

**Spots,** red spots on scapulæ, with itching and bleeding when scratched, Sumbul.

**Sprain,** pain in left scapula, as from taking cold after a sprain, in morning when turning over in bed, Rhod.

SCAPULÆ.

**Sprain,** violent stinging pain as from a pain in left scapula, extending into chest, Kali-carb.

**Sprained** pain in back and scapulæ, extending into chest two or three times a day, arresting the breath, Petrol.

— pain in right scapula on moving arm, Sul.

— pain in muscles of left shoulder and neck as if sprained, on rising from sleep at 4 P. M., Chin-ars.

— pain as from a sprain in right shoulder only on motion, Staph.

— pain in left scapula as from a sprain only on motion, Bary-c.

— pain between scapulæ and forepart of chest on moving arms, Carbo-an.

— pain as if in left scapula, Kali-c.

— pain sudden between scapulæ on lifting a weight, worse in left side with sticking upon slight movement, breathing or yawning, and on bending backward an intolerable pain, Stann.

— pain in region of left side of pelvis and between scapulæ, while at rest, with unbearable painful jerks at the slightest, Sul.

— pain in scapulæ, Nux-v.

— feeling in uppermost muscles of right scapula, on exerting the outstretched arm, Arg-m.

**Sticking** on inspiration in tip of left scapula, Lauroc.

— in inferior angles of scapulæ, Apoc.

— in shoulders when lifting arms, *Ledum.*

— in right scapula toward axilla when standing at 3 P. M., Lauroc.

— in lower end of left scapula while eating, Phelland.

— periodical sticking in scapulæ, Peonia.

— from right to left scapula, Zinc.

— in outer margin of right scapula, Cina.

— slight pain in both scapulæ, Cocc-c.

— in tip of scapulæ in mornings, Ign.

— in scapulæ, on lifting arm or anything, Iod.

SCAPULÆ.

**Sticking** in right scapula frequently, Sil.
— outward in inner surface of right scapula during rest, Sambucus.
— in lower angle of left scapula in morning, *Sul.*
— in superior angle of right scapula, Colch.
— in left scapula, Copavia, Hyos.
— in scapulæ if she works hands a little, Fer.
— in right scapula when blowing nose, Hepar.
— dull pains in shoulder joints, Staph.
— slight sticking pains in both scapulæ, Cocc-c.
— beneath right scapula while sitting, Ox-ac.
— as if something was sticking below right scapula, increased at first and disappeared after three hours' sleep, *Ars-Hyd.*
— in back, extending to right scapula, *Lyco.*
**Stiff** pain between scapulæ, Caustic.
**Stiffness** from scapula down the back, Lyco.
— in left nape and down left inner scapula, worse after waking and after laughing, *Kali-c.*
— and pain in right shoulder, especially when stretching toward the left side, Euphor.
— with pain from neck down to between scapulæ, *Apis.*
— painful in back and scapulæ, *Ledum*, Alum.
— upper scapular region a pressing pain with stiffness on sitting still, pains on beginning to move, Indium.
**Stinging**, as from blows and bruises in right scapula when in motion, Kali-c.
— posterior lateral margins of scapula, generally on left side, aching, smarting and stinging, Zizia.
— in right scapula near spine in morning, *Hyper.*
— in scapulæ, Peonia.
**Stitch** from apex of scapulæ to pit of stomach during fatiguing labor, *Kali-c.*
— dull near right scapula in back, impeding respiration, most perceptible when moving, Mez.
— dull boring in left scapula, Anac., Menyan.

SCAPULÆ.

**Stitch**, transient in left scapula and on outer side of right thigh, Bary-c.

**Stitches** and pains in left scapula, Graph.

— in right scapula, running to elbow, with burning, Cepa.

— in both scapulæ, *Phos.*

— in right scapula, *Phos.*, Tabacum.

— in left scapula during rest, worse lowering shoulder and turning trunk to left, Amm-m.

— spasmodic stitches in right scapula, worse when sitting, Ant-cr.

— dull under scapulæ, Asar.

— from chest into scapulæ, Ox-ac.

— frequent on left side of neck, extending from scapulæ to occiput, *Guaiacum.*

— intermittent on inner margin of right scapula, Ratanhia.

— in upper part of left scapula in P. M., with burning of skin in same place, Canth.

— beneath scapulæ which take away the breath, and do not permit stooping, *Sul.*

— in right scapula when blowing nose, hawking or taking deep breath, Hep.

— violent in left scapula, as from needles, Caustic.

— in scapulæ, *Sul.*

— intermittent along left scapula in morning, Sul.

— in left scapula and in cardiac region, Calc-c.

— and pressure in right scapula, *Sepia.*

— intermittent in middle of scapulæ toward spine, Stann.

— intermittent in left scapula, Verbasc.

— dull beneath right scapula, Zinc.

— sharp within scapulæ, Calc-c.

— sharp close to upper part of right scapula, most acute during eructation, Zinc.

— beneath left scapula, extending forward into left pectoral region, Zinc.

— extending into the left scapula, Zinc.

SCAPULÆ.

**Stitches** in left scapula and left breast at intervals, Mancin.
— in scapulæ while lifting something, Iod.
— dull through left scapulæ, coming out through chest in front, Bary-c.
— on inner side of scapulæ, Calc-c.
— severe lancinating pains and stitches posterior right side below scapulæ, much worse from throwing shoulders back and chest forward or any contortions of body, pain at times extorting a moan, Bad.
— under the scapulæ with outward pressing, Cepa.
**Stitchlike** pain under scapulæ while stooping, *Jug-cin.*
**Stoop,** on shoulders and between scapulæ, a drawing, tearing pain so she cannot stoop down, Borax.
**Stooping**, weakness in region of scapulæ, better by stooping, Alum.
— pain in right scapula while stooping, Cham.
— pain between scapulæ; worse on stooping and in intercostal muscles, Cham.
**Straight,** pain through from left to right shoulder, *Med.*
**Straining,** pain as if from straining from lifting, in back and scapulæ after continuous writing with the back bent, Mur-ac.
**Supper,** aching under left scapula after supper, worse by sitting, Lappa.
**Swallowing,** pressing stitching along right scapula when swallowing, hawking, or exerting voice, Caustic.
**Swollen,** as if bruised from right scapula to shoulder joint, Berb.
**Tearing** intermittent shocks on outer side of right scapula, Dulc.
— in top of right shoulder, while fingers go to sleep, awakes her at 3 A. M., Nitrum.
— in right shoulder joint, extending to scapula on pressing arm downward, Mag-m.
— on top of shoulder, down in right scapula in morning, Mag-carb.
— stitches on posterior border of right scapula, Guaiacum.

SCAPULÆ.

**Tearing** on the right side of scapula, Mez.
— in upper part of scapulæ, in bone, Merc-c.
— through scapulæ and lung, Diosc.
— in left scapula, better by friction, Phos.
— along crest of right scapula, with tension, Raphanus.
— in right scapula, Caustic., Zinc., Phos.
— on head of left scapula, worse by motion, Staph.
— beneath right scapula, Dig.
— in upper part of left scapula, when sitting, better when rising from seat, in glenoid cavity of scapula, extending to clavicle, Arg-m.
— beside the scapula, Lyco.
— in lower angle of scapulæ, from 2 P. M. till evening, when sitting still, better by moving, Alum.
— in left scapula, Thuja, Alumina.
— in right scapula, then in hip, Mag-m.
— in left scapula, when sitting, Manganum, Sul.
— in scapulæ, worse in right, Lach.
— violently in left scapula, on bending back the arm, Carbo-v.
— in right scapula, in the morning, *Kali-c.*
— severe in both scapulæ, Mag-m.
— and single tearing pains in the top of the shoulder, and pain in the left shoulder being at times so intense that she thinks she will die, Nat-carb.
— pain on left scapula, while sitting with body bent forward, Phos-ac.
— pain across scapulæ, with sticking in arms, Cyclam.
— pain between scapulæ, Anac.
— violent tearing pain between scapulæ, *Sil.*
— gnawing, shooting below scapulæ, Agari.
— pain under scapulæ when walking, Sul.

**Tension,** and pain in scapulæ and back, extending into neck, *Coloc.*
— and pain between scapulæ, *Rhus.*
— and pain between scapulæ, better by rubbing, Carbo-an.

SCAPULÆ.

**Tension**, under both scapulæ when at rest, much worse by raising arms, Con.
— and pain between scapulæ in forenoon, Alum.
— externally, near left scapula, Merc-c.
— in left scapula, as from a drawing-plaster, Lyco.
**Tensive** pain between scapulæ, when lying or moving, Sul.
— pain, with boil on right shoulder, Nitrum.
— pain above right scapula, Cicuta.
— pains in right shoulder diminished in walking, but greatly relieved by rest, Euphor.
**Thobbing** pain between scapulæ, Merc-i-fl.
— pain in right scapula, Merc-i-fl.
— painless, that ends in trembling in scapulæ, Merc.
**Tickling**, stitch in middle of right scapula, Dulc.
**Titillation** agreeable on outer border of right scapula, Dulc.
**Twinging** in lower end of left scapula, on constriction, Chellid.
**Twitching** under left scapula, Thuja.
— and rumbling in some parts of scapulæ and above right knee with contractive sensation, Rhus.
— in both scapulæ and on chest, *Calc-c.*
— in left side by scapula, when sitting, Rhus.
— pain in left scapula, extending into top of shoulder while sitting, Phos.
— and dull pain in top of shoulder as if he had borne a heavy burden, Mez.
**Tumor** on inner part of right scapula, red externally, soft in centre, *Carbo*-a.
**Ulcer**, sensation as if there was an ulcer on right scapula, Cicuta.
**Under**, aching under lower angles of right scapula, Conval.
— aching under point of left scapula, upon lifting arm, Conval.
— broad stitches as with a knife under left scapula near spinal column independent of breathing, Cup.
— scapulæ, great heat, *Elater*.
— left scapula, a twitching, Thuja.

